# Machine-Learning Approaches for Predicting the Need of Oxygen Therapy in Early-Stage COVID-19 in Japan: Multicenter Retrospective Observational Study

**DOI:** 10.3389/fmed.2022.846525

**Published:** 2022-02-23

**Authors:** Syunsuke Yamanaka, Koji Morikawa, Hiroyuki Azuma, Maki Yamanaka, Yoshimitsu Shimada, Toru Wada, Hideyuki Matano, Naoki Yamada, Osamu Yamamura, Hiroyuki Hayashi

**Affiliations:** ^1^Department of Emergency Medicine and General Internal Medicine, University of Fukui Hospital, Fukui, Japan; ^2^Connect Inc., Tokyo, Japan; ^3^Department of Emergency Medicine, Fukui Prefectural Hospital, Fukui, Japan; ^4^Department of Emergency Medicine, Tannan Regional Medical Center, Sabae, Japan; ^5^Department of Emergency Medicine, Japanese Red Cross Fukui Hospital, Fukui, Japan; ^6^Department of Emergency Medicine, Sugita Genpaku Memorial Obama Municipal Hospital, Obama, Japan; ^7^Department of Emergency Medicine, Fukui-ken Saiseikai Hospital, Fukui, Japan; ^8^Department of Community Medicine, Faculty of Medicine, University of Fukui Hospital, Fukui, Japan

**Keywords:** COVID-19, machine learning, prognostic model, medical triage, multicenter, PROBAST, TRIPOD

## Abstract

**Background:**

Early prediction of oxygen therapy in patients with coronavirus disease 2019 (COVID-19) is vital for triage. Several machine-learning prognostic models for COVID-19 are currently available. However, external validation of these models has rarely been performed. Therefore, most reported predictive performance is optimistic and has a high risk of bias. This study aimed to develop and validate a model that predicts oxygen therapy needs in the early stages of COVID-19 using a sizable multicenter dataset.

**Methods:**

This multicenter retrospective study included consecutive COVID-19 hospitalized patients confirmed by a reverse transcription chain reaction in 11 medical institutions in Fukui, Japan. We developed and validated seven machine-learning models (e.g., penalized logistic regression model) using routinely collected data (e.g., demographics, simple blood test). The primary outcome was the need for oxygen therapy (≥1 L/min or SpO_2_ ≤ 94%) during hospitalization. C-statistics, calibration slope, and association measures (e.g., sensitivity) evaluated the performance of the model using the test set (randomly selected 20% of data for internal validation). Among these seven models, the machine-learning model that showed the best performance was re-evaluated using an external dataset. We compared the model performances using the A-DROP criteria (modified version of CURB-65) as a conventional method.

**Results:**

Of the 396 patients with COVID-19 for the model development, 102 patients (26%) required oxygen therapy during hospitalization. For internal validation, machine-learning models, except for the *k*-point nearest neighbor, had a higher discrimination ability than the A-DORP criteria (*P* < 0.01). The XGboost had the highest c-statistic in the internal validation (0.92 vs. 0.69 in A-DROP criteria; *P* < 0.001). For the external validation with 728 temporal independent datasets (106 patients [15%] required oxygen therapy), the XG boost model had a higher c-statistic (0.88 vs. 0.69 in A-DROP criteria; *P* < 0.001).

**Conclusions:**

Machine-learning models demonstrated a more significant performance in predicting the need for oxygen therapy in the early stages of COVID-19.

## Introduction

The novel coronavirus disease 2019 (COVID-19), first reported in the Hubei province of the People's Republic of China in December 2019, has led to an urgent threat to global health. Many countries faced an emergency crisis despite the significant public and private efforts to delay disease spread (1). The exponential increase in COVID-19 incidence has led to a significant demand for medical services, resulting in a shortage of medical resources (2, 3). Since, it is challenging to hospitalize all patients with COVID-19 in most countries, careful observation outside the hospital is standard for asymptomatic patients and patients with mild symptoms (4). However, some asymptomatic or patients with mild symptoms of COVID-19 at the first medical visit develop severe pneumonia during observation (5, 6). Although, there is no definitive antiviral medication against COVID-19, medications, such as oxygen therapy, antiviral medication, steroids, and supportive care, have become the standard of care and have been effective (7, 8). As a result, preventable deaths occur because of the lack of adequate prediction of patient prognosis at the first medical visit and close follow-up (9). Some of them could have been saved if the need for oxygen therapy had been correctly predicted on the first visit (10). Therefore, accurate medical triage for asymptomatic patients and patients with mild symptoms in the early stage of COVID-19 is essential to decrease the incidence of death and allocate limited medical resources appropriately during the observation period (11). In previous studies, artificial intelligence, such as machine learning, has shown better predictive capabilities than traditional statistical methods (12). However, most published models have not been externally validated with calibration plots, resulting in a high risk of bias (13–15). In addition, few studies have included most patients who were hospitalized, even if the patients were asymptomatic, and have observed their progress in detail. Therefore, the performance of the reported models might be optimistic and highly biased (12, 16).

Moreover, many studies did not include a follow-up period after the end of the study. However, the need for oxygen demand and outcome may have been underestimated because the outcome of these patients may have occurred after the end of the study. With the effort for the quality required by the PROBAST (prediction model risk of bias assessment tool) guidelines and TRIPOD (transparent reporting of a multivariable prediction model for individual prognosis or diagnosis) reporting guidelines (13, 17), this study aimed to develop and validate a prognostic machine-learning model that accurately predicts the need for oxygen therapy in the early stages of COVID-19 using external datasets from multiple institutions and to compare its predictive performance with traditional approaches, such as the A-DROP criteria (consisting of age of ≥70 years in men or ≥75 years in women, blood urea nitrogen of ≥21 mg/dL or dehydration, oxyhemoglobin saturation measured by pulse oximetry of ≤ 90% or partial oxygen pressure in the arterial blood of ≤ 60 mmHg, confusion, and systolic blood pressure of ≤ 90 mmHg) (18).

## Materials and Methods

### Study Design, Source of Data, Participants Selection, and Follow Up

This multicenter retrospective cohort study analyzed patients with COVID-19 in 11 academic and community hospitals in different geographic regions across Fukui, Japan. The 11 medical institutions included two level-I and eight level-II equivalent trauma centers and a temporal medical institution. The mean annual hospitalizations are 160,000 and 90,000 in two level-I and seven level-II-equivalent trauma centers, respectively. Supplementary Table 1 and Supplementary Figure 1 show the size, location, and number of full-time physicians, hospital beds, annual outpatients, annual patients transported by ambulances, and annual hospitalized patients. The ethics review board of the University of Fukui Hospital and each participating medical institution approved the present study (approval number in the University of Fukui Hospital: 20200120). In addition, the waiver of informed consent before data collection was approved by the institutional review board of each participating medical institution. All procedures were performed according to the principles of the Declaration of Helsinki.

We retrospectively studied all consecutive patients with COVID-19 confirmed by reverse transcription-polymerase chain reaction (RT-PCR) using a pharyngeal swab test for model development and internal validation admitted to participating medical facilities. Since, the number of patients with COVID-19 in Fukui during the study period was relatively small, most patients diagnosed with COVID-19 were hospitalized and few were kept at home (<5% of all patients with COVID-19 in Fukui). We lent pulse oximeters to home care patients, instructed them on how to take measurements, and followed up with daily phone calls to check their condition. In this study, there were only a small number of patients who were initially to have mild disease and were followed up at home; hence, we could collect data closer to the actual situation.

We obtained electronic medical dataon the early stage of COVID-19 in patients admitted to 10 medical institutions (except the Ota Hospital) from July 1, 2020 to March 31, 2021 to develop prediction models and perform internal validation. This study defines medical data regarding the early stage of COVID-19 as data within 48 h of PCR confirmation or initial diagnosis. After admission to the hospital, antivirals, corticosteroids, and herbal medicines were administered by the attending physician according to the standardized methods of each medical institution.

In addition, for temporal and external validation, we retrospectively examined all consecutive patients with COVID-19 admitted to the University of Fukui Hospital, Fukui Prefectural Hospital, Japanese Red Cross Fukui Hospital, Fukui-ken Saiseikai Hospital, and Ota Hospital from April 1, 2021 to September 30, 2021.

Exclusion criteria were patients aged <17 years and pregnant patients. The present study aimed to develop and validate a machine-learning model to identify COVID-19 patients who are mildly ill at the time of initial diagnosis and who require oxygen therapy during the course of their illness. We also excluded patients with transcutaneous oxygen saturation (SpO_2_) <95%, patients receiving oxygen therapy (>1 L O_2_/min) before admission, and patients with no recorded SpO_2_ at the time of initial diagnosis.

We performed a post-discharge telephone follow-up survey on all patients 2–4 weeks after discharge to determine if any patient's condition worsened after discharge.

### Outcomes

The outcome of interest was oxygen therapy, which indicates disease progression. Oxygen therapy was administered between the time of diagnosis by PCR-positive confirmation of COVID-19 and the time of discharge from the hospital after negative confirmation by PCR. Oxygen therapy was defined as follows: (1) SpO_2_ was ≤ 94% at least once during hospitalization; (2) at least one oxygen administration of 1 L/min or more during hospitalization; (3) admission to the ICU; (4) intubation; and (5) discharge due to death. Therefore, patients who met these conditions were considered to have needed oxygen therapy.

### Data Collection and Predictors of Machine-Learning Models

From the electronic medical records of 11 medical institutions, we extracted the following routine data in the early stages of COVID-19: patient demographics [age, sex, smoking history, alcohol, height, body weight, body mass index, and comorbidities (myocardinal infarction, cognitive heart failure, peripheral vascular disease, cerebrovascular disease, chronic obstructive pulmonary disease, bronchial asthma, chronic kidney disease, hypertension, diabetes mellites, and malignancy)], symptoms (fever, fatigue, sore throat, headache, rhinorrhea, arthralgia, diarrhea, loss of smell, dyspnea, muscle ache, loss of taste, disturbance of consciousness, and conjunctival hyperemia), vital signs (systolic and diastolic blood pressure and oxygen saturation), Glasgow coma scale, complete blood count (white blood cells, lymphocytes, and platelets), coagulation profile (prothrombin time, activated partial thromboplastin time, fibrinogen, and d-dimer), biochemistry (sodium, potassium, albumin, blood urea nitrogen, creatinine, lactate dehydrogenase, aspartate aminotransferase, and alanine aminotransferase), c-reactive protein, and X-ray examination (chest X-ray or chest computed tomography). Radiology specialists or internal medicine specialists reviewed all X-ray examinations and classified them into three categories: bilateral pneumonia, unilateral pneumonia, and no pneumonia. We also recorded the medications and treatment plans after admission and outcomes.

### Statistical Analysis

We performed summary statistics to describe the characteristics of the patients and the patient's clinical course. After multiple imputations using random forests (19) (Supplementary Table 2 shows the missing rate for predictors), we preprocessed predictors, including one-shot encoding (i.e., creation of dummy variables), normalization, and standardization. In the training set (80% random sample) with all available predictors, six machine-learning models were developed: (1) penalized logistic regression (20), (2) random forest (21), (3) support vector machine (22), (4) *k*-point nearest neighbor (23), (5) XG boost (24), and (6) multilayer perceptron (25) for each outcome. In addition, we selected one machine-learning model that achieved the highest prediction performance among the six models. With the best performance model, we calculated the importance of the variables that improved the c-statistics and selected the top eight variables that improved the model performance for future practical usability. Another reason we developed the model with eight variables was to meet the PROBAST standard, which requires a large data set with at least 10 events per candidate variable for model development and at least 100 events for external validation (16). We performed stratified three-fold cross-validation to determine the optimal hyperparameters with the highest c-statistic [i.e., the area under the receiver operating characteristic (ROC) curve]. We used the A-DROP criteria, which is a modified version of CURB-65 as the reference model (26).

We measured the performance of the reference model (the A-DROP criteria) and each machine-learning model on the test set (the remaining 20% random sample) for internal validation. We estimated the c-statistics for each model and examined the relevant metrics as follows: sensitivity, specificity, positive and negative predictive values, and positive and negative likelihood ratios. Based on the ROC curve from the Youden method, we determined the threshold for perspective prediction results (cutoff) to determine the best performance model (27). We also examined the calibration plots of the best-performing model for the outcome.

For temporal external validation, we collected electronic medical record information from five hospitals. Of all the available variables, we extracted the top eight variables with missing values of 20% or less and contributed the most to improving the c-statistics using the filter method (28). We also demonstrated the c-statistics for the best machine-learning model with the eight variables and examined the relevant metrics (i.e., sensitivity), calibration plots, slope, intercepts, and coefficient of determination using temporal independent variable datasets.

A two-sided *P*-value of <0.05 was considered statistically significant. Data were analyzed using Python (version 3.7.3) and R (version 3.6.2).

## Results

### Patient Characteristics

A total of 489 patients with COVID-19 admitted to one of the 11 participating medical institutions were recorded for model development and internal validation during the 9-month study period. Of these, we excluded 28 patients aged <18 years, 14 patients with no SpO_2_ data at the first visit, and 51 patients who required oxygen administration of >1 L or SpO_2_ of <95% at the time of admission. The remaining 396 patients were included in the analytic cohort for model development.

For external validation, 855 patients were admitted to one of the five participating medical institutions during the 4 months. Of these, we excluded 90 patients under the age of 18 and 37 patients who required oxygen at the time of admission. The remaining 728 patients were included in the analytic cohort for external validation.

Patient characteristics on the admission of patients with COVID-19 are shown in Table 1. Table 2 shows the patients' vital signs, laboratory findings, X-rays on admission, and outcomes.

**Table 1:** Characteristics of the patient on the admission of the patients with COVID-19.

**Variables**	**Model developing**	**External validation**
Age, median (IQR), year	54 (34–70)	42 (27–53)
Female	192 (48)	326 (45)
Smoking history^*^	116 (32)	–
Drinking alcohol^**^	123 (52)	–
Height, median (IQR), cm	164 (157–171)	165 (158–173)
Body weight, median (IQR), kg	61 (52–72)	62 (53–73)
Body mass index, median (IQR), kg/m^2^	23 (20–25)	23 (20–25)
**Comorbidities**		
Any comorbidity	193 (49)	170 (24)
Cardiovascular all	32 (9)	–
Myocardial infarction	16 (4)	–
Congestive heart failure	5 (1)	–
Peripheral vascular disease	3 (1)	–
Cerebrovascular disease	16 (4)	–
Chronic Obstructive Pulmonary Disease (COPD)	5 (1)	–
Bronchial asthma	16 (4)	–
Chronic lung disease (excluding COPD)	4 (1)	–
Chronic kidney disease (CKD)	9 (3)	–
Hypertension	117 (30)	90 (12)
Hyperlipidemia	48 (13)	–
Diabetes mellites	52 (14)	–
Malignancy	15 (4)	–
**Symptoms**		
Any symptoms	298 (75)	–
Fever (37.0 to 38.0°C)	190 (53)	–
Fever (38.0°C or more)	47 (13)	–
Malaise or fatigue	110 (31)	–
Sore throat	92 (26)	–
Headache	60 (17)	–
Rhinorrhea	71 (20)	–
Arthralgia	37 (10)	–
Diarrhea	9 (3)	–
Loss of smell	45 (13)	–
Dyspnea	15 (4)	–
Muscle ache	37 (31)	–
Loss of taste	39 (11)	–
Disturbance of consciousness	2 (1)	–
Conjunctival hyperemia	1 (0)	–
Period from onset of symptom to PCR positive (IQR) (days)	3 (1–6)	3 (1–6)

*Data are shown as no (%) otherwise is specified*.

*Cardiovascular diseases include congestive heart failure, unstable angina pectoris, atrial fibrillation, and hypertension. Respiratory diseases include bronchial asthma, chronic obstructive pulmonary disease (COPD), tuberculosis, pleuritis, and pneumonia. Malignancy includes colonic cancer, spinal tumor, brain tumor, prostate cancer, oral cancer, and malignant lymphoma*.

*CKD, chronic kidney disease; GERD, gastroesophageal reflux disease; PCR, polymerase chain reaction*.

* *Smoking history includes patients who are currently smoking or smoking in the past*.

** *Drinking alcohol includes patients who drink daily or occasionally*.

**Table 2:** Blood pressure, percutaneous oxygen saturation, laboratory findings, X-ray on admission, and outcome of the patients with COVID-19.

**Variables**	**Model developing**	**External validation**
**Blood pressure**		
Systolic blood pressure (mmHg)	126 (115–139)	–
Diastolic blood pressure (mmHg)	83 (75–91)	–
Saturation of percutaneous oxygen (%)	97 (96–98)	98 (97–98)
**Complete blood count**		
White blood cells (×103/μL)	46 (37–56)	–
Lymphocytes (%)	25 (20–34)	–
Platelets (×104/μL)	10 (16–25)	–
**Coagulation profile**		
Prothrombin time (seconds)	11 (10–12)	–
Activated partial thromboplastin time (seconds)	32 (30–35)	–
Fibrinogen (mg/dl)	329 (247–428)	343 (290–404)
D-dimer (μg/ml)	0.8 (0.5–1.1)	0.7 (0.51–0.9)
**Biochemistry**		
Na (mEq/l)	140 (138–141)	141 (140–151)
K (mEq/l)	4 (3.8–4.2)	–
Albumin (g/dl)	4.2 (3.9–4.5)	–
Blood urea nitrogen (mg/dl)	12.6 (10.0–15.3)	–
Creatinine (mg/dl)	0.78 (0.66–0.95)	–
Lactate dehydrogenase (U/l)	194 (168–235)	
Aspartate aminotransferase (U/l)	24 (20–33)	23 (19–32)
Alanine aminotransferase (U/I)	21 (14–35)	–
**Serum**		
C-reactive protein (mg/dl)	0.45 (0.12–1.59)	0.44 (0.14–1.39)
X-ray (pneumonia)		
Non	170 (43)	382 (52)
Unilateral	42 (11)	78 (11)
Bilateral	106 (33)	244 (35)
**Outcome**		
A-DROP (≥1)	99 (25)	–
Oxygen needs	102 (26)	106 (15)

*A-DROP, Age-dehydration-respiration-orientation-blood-pressure criteria*.

The median age was 54 years [interquartile range (IQR), 34–70], and 48% of the participants were female. Overall, 102 (26%) and 106 (15%) patients needed oxygen therapy for model dataset development and external validation, respectively. A post-discharge telephone follow-up survey was performed on all patients 2–4 weeks after discharge (≥95% capture rate), and no patients were readmitted.

### Prediction Performance With all Available Variables and Important Variables

Table 3 summarizes the prediction performance of the A-DROP criteria (reference) and eight machine-learning models. Compared with the A-DROP criteria, the discrimination performance of the machine-learning models was significantly greater (*P* < 0.05), except for the *k*-point nearest-neighbor model. Among the six machine-learning models using all available variables, the XG boost had the highest c-statistic (0.89; 95% confidence interval [CI], 0.79–0.96), with a sensitivity of 0.83 (95% CI, 075–0.91), specificity of 0.90 (95% CI, 0.84–0.96), the positive predictive value of 0.71 (95% CI, 0.61–0.81), and the negative predictive value of 0.95 (95% CI, 0.90–0.99). With the filter method for variable selection, we selected the eight important variables as follows: hypertension, age, any comorbidity, aspartate aminotransferase, lactate dehydrogenase, SpO_2_ at the first visit, pneumonia, and C-reactive protein.

**Table 3:** The ability of eight machine-learning models and A-DROP as the risk stratification tool.

**Models**	**C-statistic**	***P*-value**	**Sensitivity**	**Specificity**	**PPV (95% CI)**	**NPV (95% CI)**	**PLR (95% CI)**	**NLR (95% CI)**
	**(95% CI)**		**(95% CI)**	**(95% CI)**				
A-DROP criteria ≥1 (Reference)	0.69 (0.62–0.75)	–	0.54 (0.48–0.59)	0.83 (0.79–0.87)	0.54 (0.48–0.59)	0.83 (0.79–0.87)	3.08 (2.24–4.25)	0.56 (0.41–0.77)
Penalized logistic regression	0.85 (0.75–0.95)	0.01	0.79 (0.70–0.88)	0.73 (0.64–0.82)	0.56 (0.45–0.66)	0.89 (0.82–0.95)	2.96 (1.83–4.77)	0.28 (0.18–0.46)
Random forest	0.88 (0.78–0.97)	<0.01	0.79 (0.70–0.73)	0.96 (0.93–1.00)	0.90 (0.84–0.96)	0.92 (0.85–0.97)	22.17 (5.60–7.38)	0.22 (0.05–0.86)
SVM	0.83 (0.73–0.94)	0.03	0.75 (0.65–0.84)	0.80 (0.71–0.89)	0.62 (0.51–0.75)	0.88 (0.81–0.95)	3.82 (2.14–6.81)	0.31 (0.17–0.55)
KNN	0.78 (0.66–0.91)	0.23	0.25 (0.16–0.35)	0.98 (0.95–1.00)	0.86 (0.78–0.93)	0.75 (0.66–0.85)	14.01 (1.78–110)	0.76 (0.10–6.01)
XG boost	0.89 (0.79–0.96)	<0.01	0.83 (0.75–0.91)	0.90 (0.84–0.96)	0.71 (0.61–0.81)	0.95 (0.90–0.99)	8.61 (3.92–18.9)	0.18 (0.08–0.41)
MLP	0.86 (0.76–0.96)	<0.01	0.54 (0.44–0.65)	0.95 (0.89–0.99)	0.81 (0.72–0.90)	0.83 (0.74–0.91)	10.11 (3.17–32.8)	0.48 (0.15–1.55)
XG boost with eight features (Internal validation)	0.92 (0.86–0.98)	<0.001	0.94 (0.89–0.99)	0.69 (0.59–0.79)	0.47 (0.36–0.59)	0.98 (0.94–1.00)	3.08 (2.08–4.56)	0.08 (0.05–0.12)
XG boost with eight features (External validation)	0.88 (0.81–0.95)	<0.001	0.64 (0.55–0.71)	0.93 (0.88–0.97)	0.61 (0.53–0.68)	0.93 (0.89–0.97)	8.77 (4.34–17.7)	0.39 (0.19–0.79)

*A-DROP criteria, age-dehydration-respiration-orientation-blood-pressure criteria*.

*CI, confidence interval; PPV, positive predictive value; NPV, negative predictive value; PLR, positive likelihood ratio; NLR, negative likelihood ratio; KNN, K-point nearest neighbor; MLPerceptron, multilayer perceptron; XG boost, extreme gradient boosting*.

### Prediction Performance and Feature Importance With Eight Variables

The prediction performance of the XG boost model using the eight variables is presented in Table 3. Compared with the A-DROP criteria, the discrimination performance of the XG boost models using the top eight essential variables was significantly greater (*P* < 0.001).

For internal validation, compared to the six machine-learning models using all available variables, the XG boost model using the eight variables had the highest c-statistic (0.92; 95% CI, 0.86–0.98 vs. 0.69 in A-DROP criteria; *P* < 0.001), with a sensitivity of 0.94 (95% CI, 0.89–0.99), specificity of 0.69 (95% CI, 0.59–0.79), the positive predictive value of 0.47 (95% CI, 0.36–0.59), and negative predictive value of 0.98 (95% CI, 0.94–1.00).

**Figure 1:**
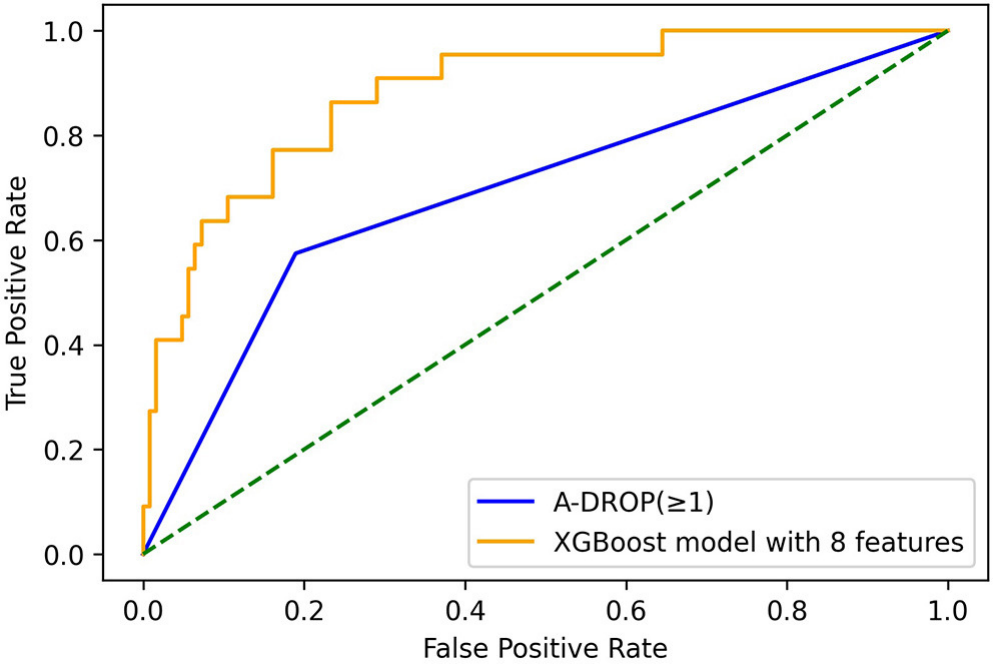
C-statistic of XG Boost model with eight variables in external validation.

For external validation using an independent data set of 728 patients, compared to the A-DROP criteria, the XG boost using the eight variables had a higher c-statistic (0.88; 95% CI, 0.81–0.95 vs. 0.69 in A-DROP criteria; *P* < 0.001; Figure 1), with a sensitivity of 0.64 (95% CI, 0.55–0.71), specificity of 0.93 (95% CI, 0.88–0.97), positive predictive value of 0.61 (95% CI, 0.53–0.68), positive predictive value of 0.93 (95% CI, 0.89–0.97]. SpO_2_ at the first visit was the most important feature, followed by age, lactate dehydrogenase, and aspartate aminotransferase levels (Table 4).

**Table 4:** Feature importance of XGboost with eight variables.

**Variables**	**Feature importance**
SpO_2_ at the first visit	0.26135576
Age	0.26132179
Lactate dehydrogenase	0.16435097
Aminotransferase	0.15464792
Any comorbidity	0.124125525
C-reactive protein	0.06478236
Hypertension	0.062515706
Pneumonia	<0.01

*SpO_2_, transcutaneous oxygen saturation*.

### Calibration Plot

**Figure 2:**
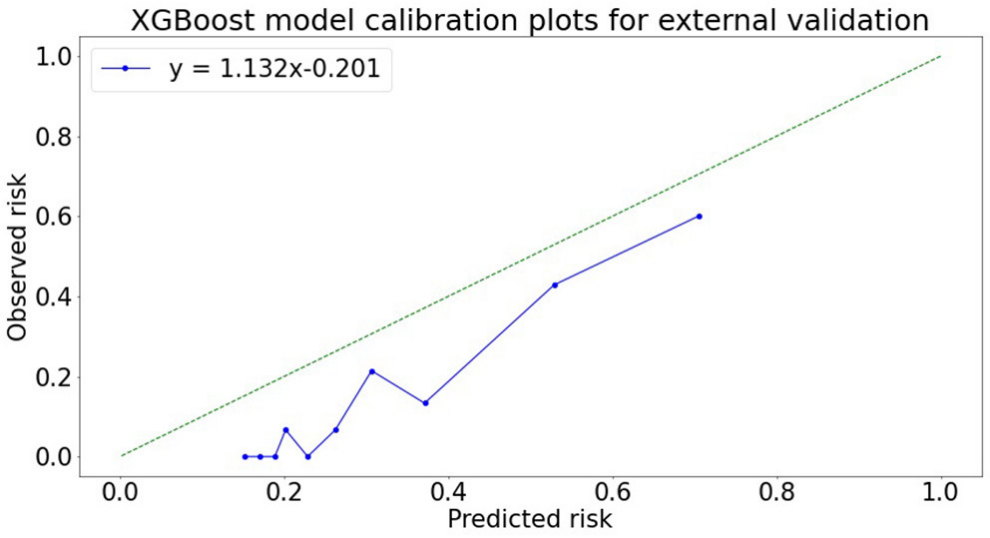
XG boost model calibration plot in external validation.

Figure 2 shows the calibration plots of the XG boost models using the eight variables for predicting outcomes for external validation. A positive relationship was observed between the predicted and actual risks in the calibration plots. The slope, intercept, and coefficient of determination of the calibration were 1.132, −0.202, and 0.949 for external validation, respectively. The model-predicted probability for external validation almost matched the observed probabilities.

## Discussion

In this study, we analyzed multicenter, retrospective data from 1,124 patients (396 for model development and internal validation, 728 for external validation) with COVID-19 and applied machine-learning models to predict oxygen therapy during the course of the infection. Specifically, most machine-learning models showed better discriminative performance than the traditional approach (i.e., the A-DROP criteria). In addition, these machine-learning models achieve a high negative predictive value in predicting outcomes, which may help us perform safer triage. To the best of our knowledge, this is the first study to investigate the performance of modern machine-learning models in predicting the need for oxygen therapy in early stage COVID-19 using a large multicenter dataset in Japan with the PROBAST standard.

It is challenging to admit all patients with COVID-19 in the current pandemic due to a lack of medical resources. The WHO guidelines recommend home treatment for patients with mild COVID-19 symptoms (29). Hence, the importance of accurate prediction for oxygen needs in the early state of COVID-19 has been emphasized, and many machine learning-based prognostic models have been developed (5, 6). However, most of their reported prediction ability may be optimistic because of the poorly reported study that did not follow PROBAST and the lack of external validation (12, 16). In addition, since these studies have looked only at hospitalized patients and not at patients considered mildly ill in the early stages of COVID-19 who were kept home without careful observation, these studies do not comprise consecutive patient data, resulting in significant selection bias (12, 16, 30). The present study examined consecutive patients admitted to 11 participating medical institutions, and few patients were kept home. We followed the PROBAST standard, which recommends an extensive data set with at least 10 events per candidate variable for model development and at least 100 events for external validation to reduce bias (31).

The XG boost model with the eight variables validated by an external dataset in the present study shows a negative predictive value of 0.93 (95% CI, 0.89–0.97). The model could contribute to a safer selection of low-risk patients at the initial diagnosis of COVID-19 and their safe management in home care under careful observation. In addition, accurate early prediction of oxygen requirements has several important implications in medical practice. For example, early identification of the potential risk of severe disease allows healthcare providers to develop individualized and optimal management strategies, prepare for hospitalization, and plan more careful follow-up.

Although the machine-learning models achieved significant predictive power, their performance remained imperfect. This can be explained, at least in part, by the limited number of predictors (e.g., the experience of healthcare professionals) and measurement errors in the data.

Furthermore, the A-DROP criterion is presumed to be simpler and easier to use. Although it is known that there is a trade-off between concise models, such as the A-DROP or CURB-65 criteria, the use of modern machine-learning models has the advantages in the era of health information technology, such as web-based applications, automated data entry through speech recognition, natural language processing, continuous model refinement through sequential extraction of electronic medical records, and reinforcement learning (32, 33). Our findings and the recent advent of machine-learning approaches collectively support cautious optimism that machine learning may enhance the clinician's ability as an assistive technology in predicting patient outcomes in the early stage of COVID-19. We have already implemented this model as a web-based application and plan to triage future COVID-19 patients in Fukui Prefecture.

This study has several limitations. First, the current model is designed to predict disease progression to the stage in which oxygen therapy is required. Hence, the present study did not attempt to predict progression to a more severe stage requiring intubation or extracorporeal membrane oxygenation. However, external validation of this study showed that four people were intubated and three of them died, but the model determined that all of them were severely ill without any omissions. Second, although the current study was conducted with a geographically diverse patient population in Fukui Prefecture, our model may not generalize to other practice settings. Therefore, this model needs to be revalidated in other parts of Japan and outside Japan. The model should be validated in other countries outside Japan. Third, the AI model should be validated repeatedly with the new COVID19 variant. Generally, every AI model should be evaluated frequently with a new dataset to maintain its prediction performance. However, we developed the AI model mainly from the alpha variant of COVID19, and the AI model performed well in external validation that primarily consisted of the delta variant. In other words, the AI model can correctly predict the severity of COVID19 even when the model predicts patients with new COVID19 variants. The AI model predicts the severity using the human body's reaction, such as CRP, not using the virus itself. Therefore, we assume if the patient's response to a new variant of COVID19 is correctly reflected in blood data and radiographs, the AI model may accurately predict the patient's severity. Finally, machine-learning models have a common limitation in terms of interpretability.

In conclusion, based on multicenter retrospective data from 1,124 patients, we developed machine-learning models to predict the need for oxygen therapy during the course of COVID-19 at its early stages. We found that, compared to conventional approaches such as the A-DROP criteria, the machine-learning models had a higher ability to predict oxygen therapy.

## Data Availability Statement

The datasets presented in this article are not readily available because we did not get permission to provide the dataset to publishers at each ethics review board of the participating hospital. Requests to access the datasets should be directed to Syunsuke Yamanaka, yamanakasyunsuke@yahoo.co.jp.

## Ethics Statement

The studies involving human participants were reviewed and approved by the Ethics Review Board of the University of Fukui Hospital. Written informed consent for participation was not required for this study in accordance with the national legislation and the institutional requirements.

## Author Contributions

SY, HA, MY, YS, TW, HM, NY OY, and HH: contributed to the conception, design of the study, and organizing the database. SY and KM: performed the statistical analysis. SY: wrote the first draft of the manuscript. All authors contributed to manuscript revision, read, and approved the submitted version.

## Funding

This study was financially supported by the health prevention division of the Fukui Prefecture.

## Conflict of Interest

KM was employed by Connect Inc. The remaining authors declare that the research was conducted in the absence of any commercial or financial relationships that could be construed as a potential conflict of interest.

## Publisher's Note

All claims expressed in this article are solely those of the authors and do not necessarily represent those of their affiliated organizations, or those of the publisher, the editors and the reviewers. Any product that may be evaluated in this article, or claim that may be made by its manufacturer, is not guaranteed or endorsed by the publisher.

## References

[1] Arabi YM Murthy S Webb S. COVID-19: a novel coronavirus and a novel challenge for critical care. Intensive Care Med. (2020) 46:833–6. 10.1007/s00134-020-05955-1 32125458 PMC7080134

[2] Grasselli G Pesenti A Cecconi M. Critical care utilization for the COVID-19 outbreak in Lombardy, Italy: early experience and forecast during an emergency response. JAMA. (2020) 323:1545–6. 10.1001/jama.2020.4031 32167538

[3] Remuzzi A Remuzzi G. COVID-19 and Italy: what next? Lancet. (2020) 395:1225–8. 10.1016/S0140-6736(20)30627-9 32178769 PMC7102589

[4] Gao Z Xu Y Sun C Wang X Guo Y Qiu S. A systematic review of asymptomatic infections with COVID-19. J Microbiol Immunol Infect. (2021) 54:12–6. 10.1016/j.jmii.2020.05.001 32425996 PMC7227597

[5] Tabata S Imai K Kawano S Ikeda M Kodama T Miyoshi K. Clinical characteristics of COVID-19 in 104 people with SARS-CoV-2 infection on the Diamond Princess cruise ship: a retrospective analysis. Lancet Infect Dis. (2020) 20:1043–50. 10.1016/S1473-3099(20)30482-5 32539988 PMC7292609

[6] Zhou F Yu T Du R Fan G Liu Y Liu Z. Clinical course and risk factors for mortality of adult inpatients with COVID-19 in Wuhan, China: a retrospective cohort study. Lancet. (2020) 395:1054–62. 10.1016/S0140-6736(20)30566-3 32171076 PMC7270627

[7] Cui Y Sun Y Sun J Liang H Ding X Sun X. Efficacy and safety of corticosteroid use in coronavirus disease 2019 (COVID-19): a systematic review and meta-analysis. Infect Dis Ther. (2021) 10:2447–63. 10.1007/s40121-021-00518-3 34389970 PMC8363240

[8] Nangaku M Kadowaki T Yotsuyanagi H Ohmagari N Egi M Sasaki J. The Japanese Medical Science Federation COVID-19 Expert Opinion English Version. JMA J. (2021) 4:148–62. 10.31662/jmaj.2021-0002 33997449 PMC8118966

[9] Branas CC Rundle A Pei S Yang W Carr BG Sims S. Flattening the curve before it flattens us: hospital critical care capacity limits and mortality from novel coronavirus (SARS-CoV2) cases in US counties. medRxiv. (2020). 10.1101/2020.04.01.20049759

[10] Sanders JM Monogue ML Jodlowski TZ Cutrell JB. Pharmacologic treatments for coronavirus disease 2019 (COVID-19): a review. JAMA. (2020) 323:1824–36. 10.1001/jama.2020.6019 32282022

[11] Lee YH Hong CM Kim DH Lee TH Lee J. Clinical course of asymptomatic and mildly symptomatic patients with coronavirus disease admitted to community treatment centers, South Korea. Emerg Infect Dis. (2020) 26:2346–52. 10.3201/eid2610.201620 32568662 PMC7510714

[12] Shamsoddin E. Can medical practitioners rely on prediction models for COVID-19? A systematic review. Evid Based Dent. (2020) 21:84–6. 10.1038/s41432-020-0115-5 32978532 PMC7517064

[13] Wolff RF Moons KGM Riley RD Whiting PF Westwood M Collins GS. PROBAST: a tool to assess the risk of bias and applicability of prediction model studies. Ann Intern Med. (2019) 170:51–8. 10.7326/M18-1376 30596875

[14] Najafabadi ZAH Ramspek CL Dekker FW Heus P Hooft L Moons KGM. TRIPOD statement: a preliminary pre-post analysis of reporting and methods of prediction models. BMJ Open. (2020) 10:e041537. 10.1136/bmjopen-2020-041537 32948578 PMC7511612

[15] Collins GS Reitsma JB Altman DG Moons KG. Transparent reporting of a multivariable prediction model for individual prognosis or diagnosis (TRIPOD): the TRIPOD statement. BMJ. (2015) 350:g7594. 10.1136/bmj.g7594 25569120

[16] Wynants L Van Calster B Collins GS Riley RD Heinze G Schuit E. Prediction models for diagnosis prognosis of covid-19: systematic review critical appraisal. BMJ. (2020) 369:m1328. 10.1136/bmj.m1328 32265220 PMC7222643

[17] Moons KG Altman DG Reitsma JB Ioannidis JP Macaskill P Steyerberg EW. Transparent reporting of a multivariable prediction model for individual prognosis or diagnosis (TRIPOD): explanation and elaboration. Ann Intern Med. (2015) 162:W1–W73. 10.7326/M14-0698 25560730

[18] Miyashita N Matsushima T Oka M. The JRS guidelines for the management of community-acquired pneumonia in adults: an update and new recommendations. Intern Med. (2006) 45:419–28. 10.2169/internalmedicine.45.1691 16679695

[19] Stekhoven DJ. Nonparametric Missing Value Imputation Using Random Forest. R package version 1.4 (2012).32380076

[20] Warton DI. Penalized normal likelihood and ridge regularization of correlation and covariance matrices. J Am Stat Assoc. (2008) 103:340–9. 10.1198/016214508000000021

[21] Svetnik V Liaw A Tong C Culberson JC Sheridan RP Feuston BP. Random forest: a classification and regression tool for compound classification and QSAR modeling. J Chem Inf Comput Sci. (2003) 43:1947–58. 10.1021/ci034160g 14632445

[22] Suykens JA Vandewalle J. Least squares support vector machine classifiers. Neural Process Lett. (1999) 9:293–300. 10.1023/A:101862860974211972910

[23] Bay SD. Nearest neighbor classification from multiple feature subsets. Intell Data Anal. (1999) 3:191–209. 10.1016/S1088-467X(99)00018-9

[24] Chen T He T Benesty M Khotilovich V Tang Y. Xgboost: Extreme Gradient Boosting. R package version 0.4-2. 1-4 (2015).

[25] Pal S Mitra S. Multilayer perceptron, fuzzy sets, and classification. IEEE Trans Neural Netw. (1992) 3:683–97. 10.1109/72.159058 18276468

[26] Shindo Y Sato S Maruyama E Ohashi T Ogawa M Imaizumi K. Comparison of severity scoring systems A-DROP and CURB-65 for community-acquired pneumonia. Respirology. (2008) 13:731–5. 10.1111/j.1440-1843.2008.01329.x 18713094

[27] Tolga T Jiju A. The assessment of quality in medical diagnostic tests: a comparison of ROC/Youden and Taguchi methods. Int J Health Care Qual Assur. (2000) 13:300–7. 10.1108/09526860010378744 11484648

[28] Guyon I Elisseeff A. An introduction to variable and feature selection. J Mach Learn Res. (2003) 3:1157–82. 10.1162/153244303322753616

[29] World Health Organization. Home Care for Patients With COVID-19 Presenting With Mild Symptoms and Management of Their Contacts: Interim Guidance (2020). Available online at: https://www.who.int/publications/i/item/home-care-for-patients-with-suspected-novel-coronavirus-(ncov)-infection-presenting-with-mild-symptoms-and-management-of-contacts (accessed March 17, 2020).

[30] Luo M Liu J Jiang W Yue S Liu H Wei S. IL-6 and CD8+ T cell counts combined are an early predictor of in-hospital mortality of patients with COVID-19. JCI Insight. (2020) 5:e139024. 10.1172/jci.insight.139024 32544099 PMC7406244

[31] Collins GS Ogundimu EO Altman DG. Sample size considerations for the external validation of a multivariable prognostic model: a resampling study. Stat Med. (2016) 35:214–26. 10.1002/sim.6787 26553135 PMC4738418

[32] Freeman MB,. Method Apparatus for Automated Data Entry. Google Patents (2001). Available online at: https://patents.justia.com/patent/6299063 (accessed January 14, 2022).

[33] Thanaki J. Python Natural Language Processing. Birmingham: Packt Publishing Ltd. (2017).

