# Supplementary material for: Machine-Learning Approaches for Predicting the Need of Oxygen Therapy in Early-Stage COVID-19 in Japan: Multicenter Retrospective Observational Study
Source: Front Med (Lausanne). 2022 Feb 23;9:846525. doi: 10.3389/fmed.2022.846525 (PMC8904892; doi:10.3389/fmed.2022.846525)
Supplement: Supplementary file 1 [file Table_1.docx]

Supplemental table1: The scale of participating medical institution

| No | Hospital name | Level  of center | Full-time doctors | Bet | Annual outpatients | Ambulancesper year | Hospitalizations per year |
| --- | --- | --- | --- | --- | --- | --- | --- |
| 1 | University of Fukui Hospital | level-Ⅱ | 366 | 600 | 524,140 | 2,000 | 193,085 |
| 2 | Fukui Prefectural Hospital | level-Ⅰ | 178 | 809 | 247,192 | 3,470 | 183613 |
| 3 | Japanese Red Cross Fukui Hospital | level-Ⅱ | 158 | 534 | 250,163 | 2,922 | 141,977 |
| 4 | Fukui-ken Saiseikai Hospital | level-Ⅱ | 150 | 460 | 250,000 | 2,000 | 140,000 |
| 5 | Tannan Regional Medical Center | level-Ⅱ | 27 | 179 | 127,225 | 1,207 | 39,665 |
| 6 | Municipal Tsuruga Hospital | level-Ⅱ | 65 | 332 | 152,630 | 2,044 | 5,040 |
| 7 | Sugita Genpaku Memorial Obama Municipal Hospital | level-Ⅰ | 58 | 456 | 172,526 | 1,719 | 121426 |
| 8 | Fukui Katsuyama General Hospital | level-Ⅱ | 27 | 199 | 115,501 | 933 | 57,930 |
| 9 | Nakamura Hospital | level-Ⅱ | 17 | 199 | 96,688 | 1296 | 51130 |
| 10 | Fukui City Children's Outdoor Learning Center | Unspecified | non-disclosure | non-disclosure | non-disclosure | non-disclosure | non-disclosure |
| 11 | Echizen Town Public Health Insurance Ota Hospital | level-Ⅱ | 7 | 55 | non-disclosure | 600 | non-disclosure |
