# Supplementary material for: Machine-Learning Approaches for Predicting the Need of Oxygen Therapy in Early-Stage COVID-19 in Japan: Multicenter Retrospective Observational Study
Source: Front Med (Lausanne). 2022 Feb 23;9:846525. doi: 10.3389/fmed.2022.846525 (PMC8904892; doi:10.3389/fmed.2022.846525)
Supplement: Supplementary file 2 [file Table_2.DOCX]

**Supplemental Table 1. The proportion of missingness in predictors and outcomes used in machine learning models.**

| **Predictors** | **Model developing** | **External**  **validation** |
| --- | --- | --- |
| Age | 1 (0) | 0 (0) |
| Female | 0 (0) | 0 (0) |
| Smoking history* | 42 (11) | - |
| Drinking alcohol** | 162 (41) | - |
| Height | 19 (5) | 10 (1) |
| Body weight | 16 (4) | 6 (1) |
| Body mass index | 21 (5) | 8 (-) |
| Comorbidities |  |  |
| Any comorbidity | 42 (11) | 1 (0) |
| Cardiovascular all | 42 (11) | - |
| Myocardial infarction | 42 (11) | - |
| Congestive heart failure | 42 (11) | - |
| Peripheral vascular disease | 42 (11) | - |
| Cerebrovascular disease | 42 (11) | - |
| Chronic Obstructive Pulmonary Disease (COPD) | 42 (11) | - |
| Bronchial asthma | 42 (11) | - |
| Chronic lung disease (excluding COPD) | 42 (11) | - |
| Chronic kidney disease (CKD) | 42 (11) | - |
| Kidney disease (excluding CKD) | 42 (11) | - |
| Hypertension | 0 (0) | 1 (0) |
| Hyperlipidemia | 42 (11) | - |
| Diabetes mellites | 42 (11) | - |
| Malignancy | 42 (11) | - |
| Symptoms |  |  |
| Any symptoms | 1 (0) | - |
| Fever (37.0℃ to 38.0 ℃) | 42 (11) | - |
| Fever (38.0℃ or more) | 42 (11) | - |
| Malaise or fatigue | 42 (11) | - |
| Sore throat | 42 (11) | - |
| Headache | 42 (11) | - |
| Rhinorrhea | 42 (11) | - |
| Arthralgia | 42 (11) | - |
| Chill | 42 (11) | - |
| Nasal obstruction | 42 (11) | - |
| Throat discomfort | 42 (11) | - |
| Loss of smell | 42 (11) | - |
| Diarrhea | 42 (11) | - |
| Muscle ache | 42 (11) | - |
| Sputum | 42 (11) | - |
| Loss of taste | 42 (11) | - |
| Anorexia | 42 (11) | - |
| Nausea or vomiting | 42 (11) | - |
| Dyspnea | 42 (11) | - |
| Abdominal pain | 42 (11) | - |
| Chest pain | 42 (11) | - |
| Conjunctival hyperemia | 42 (11) | - |
| Period from onset of symptom to PCR positive | 84 (22) | 0 (0) |
| Blood pressure |  |  |
| Systolic blood pressure | 52 (13) | - |
| Diastolic blood pressure | 76 (19) | - |
| Saturation of percutaneous oxygen (SpO2) (%) | 0 (0) | 0 (0) |
| Complete blood count |  |  |
| White blood cells (×103/μL) | 53 (13) | - |
| Lymphocytes (×/μL) | 72 (18) | - |
| Platelets (×104/μL) | 52 (13) | - |
| Coagulation profile |  |  |
| Prothrombin time-international normalized ratio (PT-INR) | 197 (49) | - |
| Activated partial thromboplastin time (APTT) | 247 (62) | - |
| Fibrinogen (mg/dl) | 187 (47) | 178 (35) |
| D-dimer (μg/ml) | 144 (36) | 256 (24) |
| Biochemistry |  |  |
| Na (mEq/l) | 37 (9) | 32 (4) |
| K (mEq/l) | 74 (18) | - |
| Albumin (g/dl) | 80 (20) | - |
| Blood urea nitrogen (mg/dl) | 64 (16) | - |
| Creatinine (mg/dl) | 25 (6) | 32 (4) |
| Lactate dehydrogenase (LDH) (U/l) | 38 (9) | 30 (4) |
| Aspartate aminotransferase (AST) (U/l) | 33 (8) | 30 (4) |
| Alanine aminotransferase (ALT) (U/I) | 72 (18) | - |
| Serum |  |  |
| C-reactive protein (CRP) (mg/dl) | 25 (6) | 37 (4) |
| X-ray (Pneumonia) | 71 (18) | 25 (3) |
| Outcome |  |  |
| ADROP≥1 | 32 (8) | - |
| Oxygen needs | 0 (0) | 0 (0) |

Data were shown as no (%) otherwise is specified.

Cardiovascular diseases include congestive heart failure, unstable angina pectoris, atrial fibrillation, and hypertension. Respiratory diseases include bronchial asthma, chronic obstructive pulmonary disease (COPD), tuberculosis, pleuritis, and pneumonia. Malignancy includes colonic cancer, spinal tumor, brain tumor, prostate cancer, oral cancer, and malignant lymphoma. CKD: Chronic kidney disease; GERD: gastroesophageal reflux disease; PCR: polymerase chain reaction

* Smoking history includes the patients who are currently smoking or smoking in the past.

** Drinking alcohol includes the patients who drink daily or occasionally.
