# Supplementary material for: Machine-Learning Approaches for Predicting the Need of Oxygen Therapy in Early-Stage COVID-19 in Japan: Multicenter Retrospective Observational Study
Source: Front Med (Lausanne). 2022 Feb 23;9:846525. doi: 10.3389/fmed.2022.846525 (PMC8904892; doi:10.3389/fmed.2022.846525)
Supplement: Supplementary file 3 [file Presentation_1.pptx]

## Slide 1
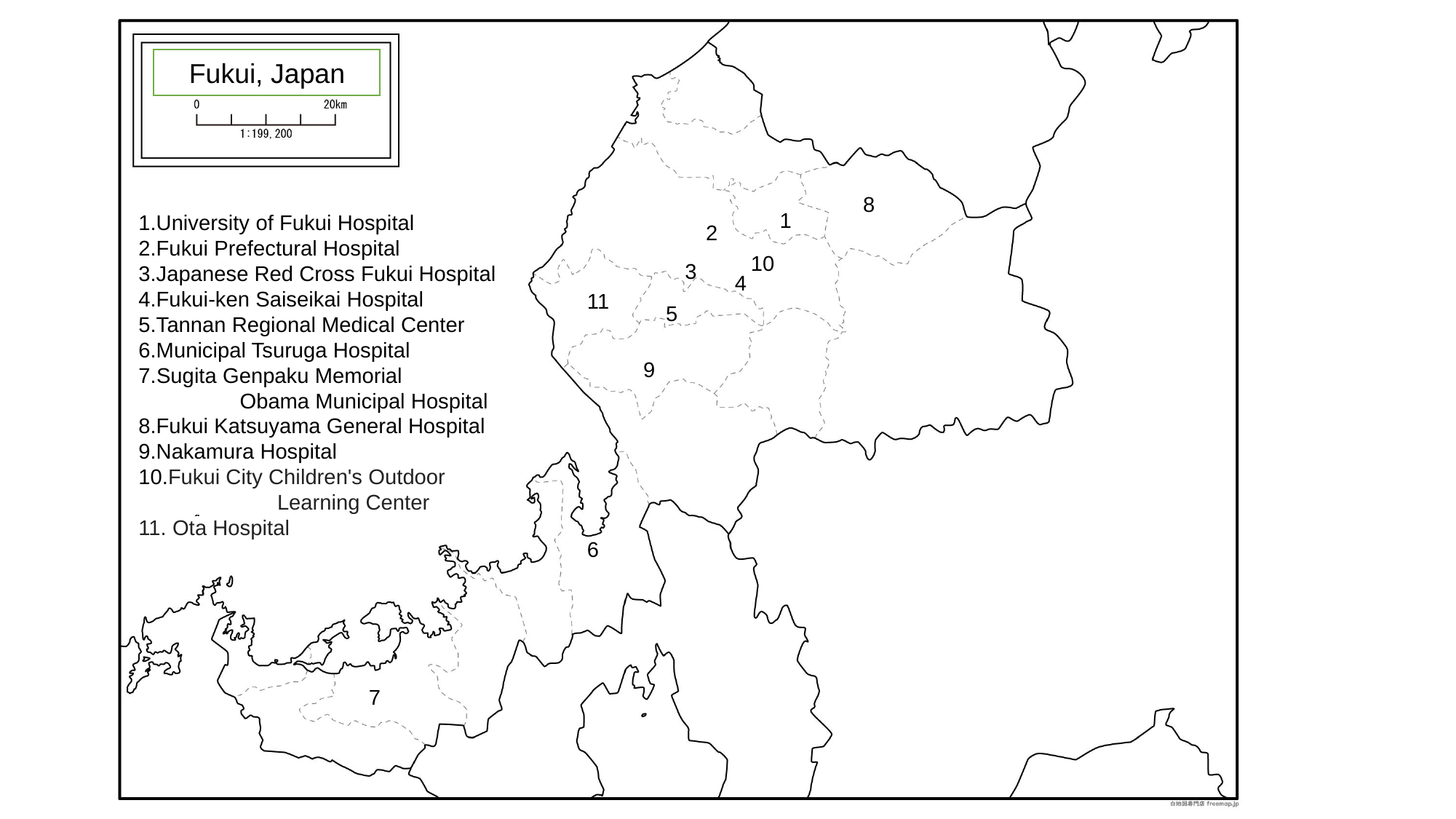

Fukui, Japan
8
1
1.University of Fukui Hospital
2.Fukui Prefectural Hospital
3.Japanese Red Cross Fukui Hospital
4.Fukui-ken Saiseikai Hospital
5.Tannan Regional Medical Center
6.Municipal Tsuruga Hospital
7.Sugita Genpaku Memorial
 　　　　Obama Municipal Hospital
8.Fukui Katsuyama General Hospital
9.Nakamura Hospital
10.Fukui City Children's Outdoor
　　　　　　 Learning Center
11. Ota Hospital
2
10
3
4
11
5
9
6
7
